# Family physicians’ acceptability, perceived value, and barriers of pharmacist-led medication management in primary care—a theory-based qualitative study

**DOI:** 10.3389/fmed.2026.1949962

**Published:** 2026-10-28

**Authors:** Zeynep İpek Salıcı, Robin Sertkaya, Çağlar Macit, Mesut Sancar, Betul Okuyan, Abdikarim Mohamed Abdi

**Affiliations:** 1 Department of Clinical Pharmacy, Graduate School of Health Sciences, İstanbul Medipol University, Istanbul, Türkiye; 2 Department of Clinical Pharmacy, Yeditepe University, Istanbul, Türkiye; 3 Department of Pharmacology, School of Pharmacy, İstanbul Medipol University, Istanbul, Türkiye; 4 Department of Clinical Pharmacy, Marmara University, Istanbul, Türkiye

**Keywords:** family physicians, comprehensive medication management (CMM), pharmacists, primary health care, qualitative research, Türkiye

## Abstract

**Background:**

Polypharmacy burdens healthcare systems and increases adverse drug events. While pharmacist-led comprehensive medication management (CMM) mitigates these issues globally, its integration into Turkish primary care remains limited.

**Objective:**

To explore family physicians’ acceptability of pharmacist-led CMM in Türkiye and identify perceived barriers to interprofessional collaboration.

**Methods:**

A qualitative descriptive study was conducted among primary care family physicians in Istanbul, Türkiye, between June and August 2025. Purposive sampling was utilized to recruit participants with at least 5 years of clinical experience. We collected data through semi-structured online interviews using a guide developed by an interdisciplinary panel and piloted for structural clarity. With NVivo software, transcripts were analyzed using a hybrid thematic analysis approach, combining inductive coding to identify emergent, context-specific themes with deductive mapping to the seven constructs of the Theoretical Framework of Acceptability (TFA). Inter-coder reliability was established through independent parallel coding, and trustworthiness was reinforced via member checking. Reporting adhered to the COREQ checklist.

**Results:**

Participants (*n* = 12) facing severe daily workloads (>74 patients) viewed clinical pharmacists as a crucial safety net. Analysis revealed three core themes: (1) *Needs and Opportunities:* CMM offers substantial benefits by managing polypharmacy, mitigating physician fatigue, reinforcing patient medication adherence, and reducing overall workload and costs. (2) *Concerns and Barriers:* Major structural hurdles include physical space constraints in FHCs, data privacy (KVKK) vulnerabilities, and pharmaceutical industry apprehension. (3) *Implementation Conditions:* Successful integration requires integrated Electronic Health Records (EHR), formalized legal boundaries, and joint interprofessional training. Subgroup analysis indicated that early- to mid-career physicians demonstrated higher implementation readiness, while late-career peers focused more on operational burdens.

**Conclusion:**

Family physicians demonstrate highly favorable prospective acceptability toward pharmacist-led CMM. Successful real-world integration requires resolving macro-level infrastructural and legal barriers while establishing actionable interprofessional enablers, shared clinical algorithms, and compatible digital workflows.

## Graphical Abstract

**Figure:**
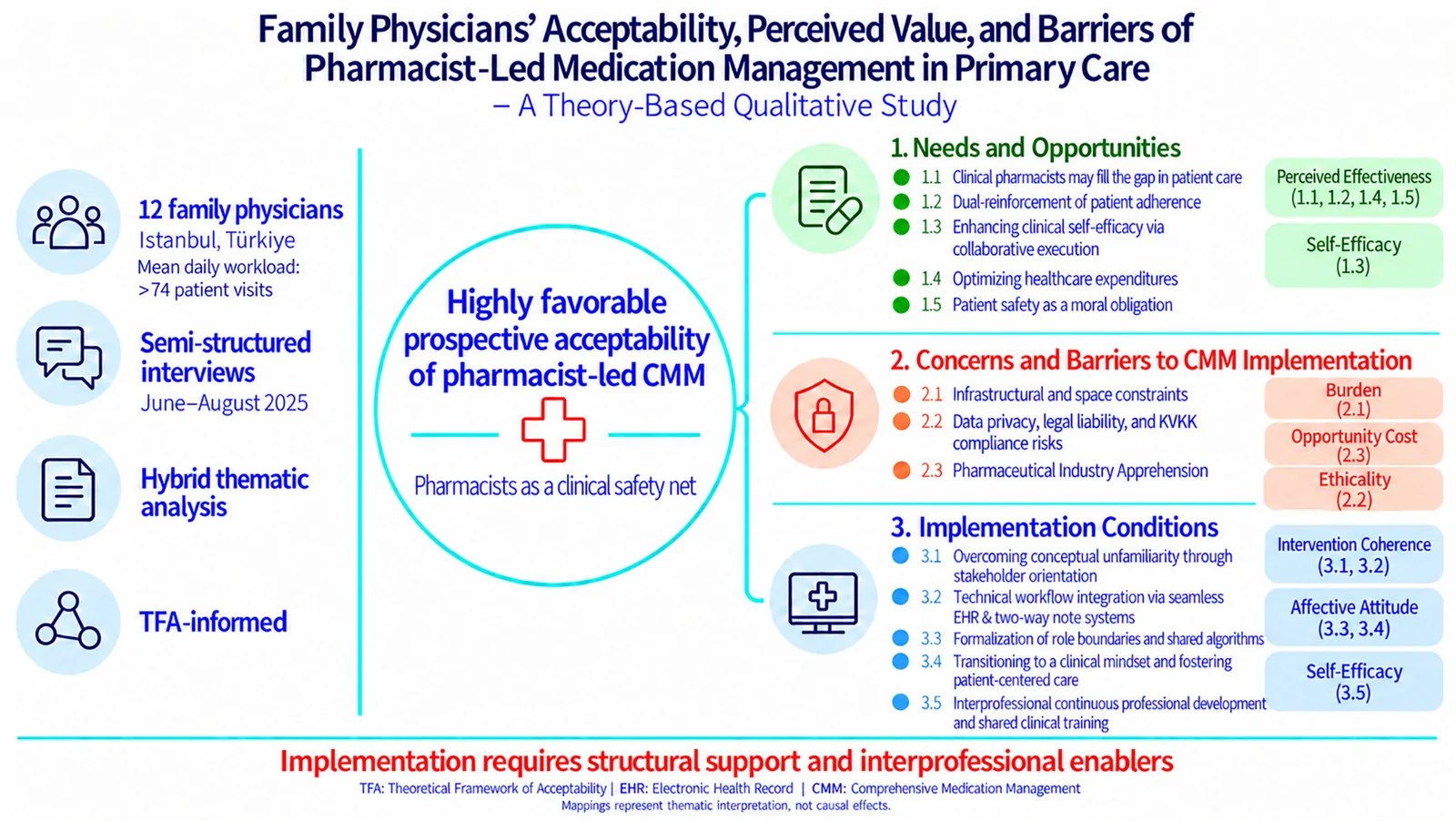

## Introduction

The WHO operational framework for primary health care (2020) highlights that reinforcing Primary Health Care (PHC) is essential for building sustainable health systems, achieving Universal Health Coverage (UHC), and reaching health-related Sustainable Development Goals (1). However, the global rise in multimorbidity and population aging has triggered a “polypharmacy epidemic,” with more patients than ever being prescribed five or more long-term medications (2). While the clinical risks of polypharmacy—including adverse drug events (ADEs) and preventable hospitalizations—are well documented (3), its impact on the healthcare workforce is equally critical. For family physicians, polypharmacy imposes a profound professional burden marked by high cognitive load, complex decision-making, and “clinical inertia,” underscoring the urgent need for structural intervention at the primary care level (4, 5).

In healthcare systems across the United Kingdom, Canada, and Australia, pharmacist-led Comprehensive Medication Management (CMM) has consistently reduced the physician’s medication-related workload by delegating complex titration and deprescribing to pharmacists (6–9). CMM is a collaborative, patient-centered approach in which a clinical pharmacist reviews a patient's entire medication regimen to ensure each drug is appropriate, effective, and safe, and to support patient medication adherence. By optimizing therapeutic outcomes, CMM enables family physicians to reclaim clinical time, thereby improving patient safety and clinician well-being (10). In these global contexts, clinical pharmacists work under formal collaborative practice agreements, have direct access to shared Electronic Health Record (EHR) systems, and follow structured payment models.

In Türkiye, exploring such collaborative models is increasingly urgent as the country undergoes a rapid demographic shift. By 2024, the population aged 65 and over reached 9.1 million (10.6% of the total population)—a 20.7% increase in just five years (11). This aging trend is accompanied by a heavy burden of non-communicable diseases (NCDs); circulatory system diseases accounted for 34.7% of all deaths in 2025, with ischemic heart diseases representing the leading sub-cause at 42.3% (12), and diabetes prevalence has surged to 14.5% (13). As a result, polypharmacy affects up to 58.3% of elderly patients in Turkish primary care (14), with multimorbid individuals facing a ninefold increase in medication-related risks (15).

Despite these challenges, the Turkish “Family Medicine Model” within Family Health Centers (FHCs) remains physician-centric and under-resourced (16, 17). With only 2.4 practicing doctors per 1,000 people (well below the OECD average of 3.9) (16), family physicians manage a staggering 80–100 patients daily, leaving little or no time for comprehensive consultations or systematic screening for chronic diseases (17). Furthermore, unlike the integrated systems in the UK or North America, community pharmacists in Türkiye operate as independent retail providers without direct clinical integration into FHCs, interoperable health software, or institutional pathways for medication review services. Given these structural disparities, evaluating family physicians' prospective acceptability, perceived value, and implementation concerns is an essential prerequisite before introducing pharmacist-led CMM into Turkish primary care.

Although Türkiye formally recognized clinical pharmacy as a specialty in 2014 under Law No. 6514 and began appointing its first cohort of clinical pharmacy specialists to state hospitals in 2022–2023, these experts remain confined to inpatient hospital settings and are entirely absent from FHC settings (18, 19). The resulting “implementation gap” is profound: although specialized human resources are now available, the system lacks formal legislative frameworks and official job descriptions to integrate clinical pharmacists into the multidisciplinary primary care team.

The transition toward interprofessional collaboration is a behavioral and social challenge. By grounding research in established behavioral theories—as recommended by the European Society of Clinical Pharmacy for pharmacist involvement—researchers can ensure that new collaborative models are both evidence-based and feasible in high-pressure clinical settings (20). In countries with historically physician-led hierarchies, new interprofessional roles can provoke professional resistance, especially when scope and boundaries are unclear; however, acceptance by dominant prescribing stakeholders strongly influences adoption and sustainability across governance, leadership, training, role clarity, and resourcing (21, 22).

The Theoretical Framework of Acceptability (TFA) conceptualizes acceptability across seven distinct component constructs:

- *Affective Attitude:* How an individual feels about the intervention.
- *Burden:* The perceived amount of effort required to participate.
- *Ethicality:* The extent to which the intervention fits with an individual’s value system.
- *Intervention Coherence:* The participant’s understanding of the intervention and how it works.
- *Opportunity Costs:* The extent to which benefits or values must be given up when engaging in the intervention.
- *Perceived Effectiveness:* The extent to which the intervention is perceived as likely to achieve its purpose.
- *Self-efficacy:* The participant’s confidence in their ability to perform the behaviors required for the intervention (22).

Although the international literature provides extensive data on CMM within health systems in a limited number of countries, it remains entirely unknown how Turkish family physicians perceive the potential introduction of an unfamiliar clinical specialist into their high-pressure, high-volume FHC settings. Therefore, this study aims to systematically evaluate, from family physicians' perspectives, the prospective acceptability of integrating pharmacist-led CMM into Turkish primary care, using the TFA to identify context-specific barriers and inform future workforce policies and collaborative frameworks within FHCs.

## Methods

### Study design and setting

This research employed a qualitative descriptive design to explore the acceptability of integrating clinical pharmacists into primary care as CMM providers. The study was conducted in FHCs located across Istanbul, Türkiye, which operate under the national Family Medicine Model. At the time of the study, clinical pharmacists were not formally integrated or employed within these primary care facilities.

### Participants, sampling, and recruitment

Purposive sampling was used to recruit family physicians and capture a diverse range of perspectives on CMM integration. To be eligible for inclusion, participants had to be currently practicing under the Turkish FHC framework and have at least 5 years of clinical experience in primary care. To ensure broad representation, the sample included family physicians from 5 Family Health Centers across three socio-economically and demographically distinct districts in Istanbul: Kadıköy (high-income/aging), Ümraniye (mixed/developing), and Güngören (low-income/dense). We selected these districts to capture the city's diverse structural contexts.

We recruited participants through direct outreach and administrative visits to FHCs. Recruitment and data analysis proceeded in parallel. To respect participants’ time and minimize research fatigue among a heavily burdened healthcare workforce, sampling was stopped when thematic saturation was reached. Saturation was operationalized using a two-layered approach: *code saturation* (the point at which no new discrete conceptual tags emerge) and *meaning saturation* (the stage at which the properties, dimensions, and operational nuances of established codes are fully elaborated). The research team established an *a priori* stopping rule that required three consecutive interviews with no new code generation to declare codebook stabilization. Code saturation was achieved early in the analytical cycle, and meaning saturation Because Interviews 10, 11, and 12 revealed substantial contextual variation but no new conceptual codes, data collection was formally closed after 12 interviews. 12 yielded deep contextual variations but no new conceptual codes; data collection was formally closed after 12 interviews. The remaining scheduled participants (Physicians 13–15) were formally released, with expressions of gratitude. The two physicians who participated in the initial pilot study were excluded from the final analytical sample.

### Theoretical framework and interview guide

Data collection followed a semi-structured interview guide developed around the seven domains of the Theoretical Framework of Acceptability (TFA): *affective attitude, burden, ethicality, intervention coherence, opportunity costs, perceived effectiveness, and self-efficacy* (22). We adopted the TFA because collaborative CMM represents a novel, prospective intervention in Turkish primary care, making prospective acceptability evaluations imperative to delineate systemic, cognitive, and structural barriers before formal rollout (23). Because CMM involves interprofessional execution, we adapted the self-efficacy construct from its strict single-actor definition to capture physicians' confidence in collaborative workflows and in executing shared clinical tasks (24).

An interdisciplinary panel of two clinical pharmacists, an academic pharmacy practice research expert, and a practicing family physician developed the interview guide (see Supplementary Material S2). To ensure a uniform understanding among participants before the evaluation, CMM was operationally described during the interviews (Guide Item 3) as a collaborative pharmacist-physician service that prioritizes multimorbid and polypharmacy patients and encompasses disease management, regular review of drug therapy, prevention of drug-related problems, and medication adherence support. We adapted the questions from validated TFA implementation instruments (22–25) and tailored them to the operational context of Turkish FHCs. To ensure methodological rigor, readability, and conceptual clarity, we conducted a pilot study with two family physicians. The pilot data confirmed the questions' accessibility, and minor phrasing adjustments were made before full data collection.

### Data collection and interpretation

Data were collected between June and August 2025 through semi-structured Zoom interviews. Interviews lasted an average of 23 min (range: 13–37 min). To ensure consistency and rigor, the primary investigators (ZIS and RS) underwent intensive qualitative research training facilitated by a senior expert in pharmacy practice research.

The lead researcher (ZIS), a clinical pharmacist and PhD candidate, transcribed the audio recordings verbatim in Turkish and then translated them into English. Field notes were not taken after interviews to capture context. Recognizing the inherent interpretative cost of analyzing translated qualitative data, we used a rigorous bilingual analytical approach to prevent loss of meaning or cultural nuance. We analyzed the data using NVivo Version 1.7.1 (Lumivero, Denver, CO, USA), employing a hybrid thematic analysis approach (26). This involved an iterative three-stage process:

1. Primary deductive coding using a codebook pre-defined by the seven domains of the Theoretical Framework of Acceptability (TFA);
2. Inductive coding of transcripts to capture context-specific, emergent nuances unique to the Turkish primary care landscape; and
3. Clustering these codes into three overarching themes (Needs and Opportunities, Concerns/Barriers, and Conditions) and mapping their subthemes back to the relevant TFA dimensions.

To ensure inter-coder reliability, one investigator (AA) independently analyzed and coded the English transcripts, while the primary investigator (ZIS) simultaneously coded the original Turkish transcripts. Following this parallel independent analysis, both researchers (ZIS and AA) convened to evaluate, compare, and synthesize the identified themes. Any discrepancies were resolved through iterative consensus, ensuring that the final framework accurately captured the participants' original sentiments without translation-induced bias. We reinforced trustworthiness through independent parallel bilingual coding, member checking, iterative consensus meetings among researchers, and a detailed audit trail of analytical decisions (27).

To explore how prospective acceptability might vary across career trajectories—critical for tailoring future implementation strategies—we conducted a subgroup pattern analysis using the Framework Method (28). By mapping thematic variations across participant characteristics, we systematically identified diverging nuances in prospective acceptability based on physicians' clinical experience.

### Ethical considerations

The Istanbul Medipol University Non-Interventional Clinical Research Ethics Committee approved the study protocol (Decision No: E-10840098-202.3.02-3977) on July 5, 2024. The ethics committee approval explicitly covered remote interviews via Zoom and the audio recording of these sessions. All participants provided recorded verbal informed consent before interviews. To protect anonymity, we removed all identifying information during transcription and assigned participants numerical identifiers (e.g., Physician 1–12). The study followed the ethical principles of the Declaration of Helsinki.

## Results

### Demographic profile and contextual indicators

Out of 22 invited family physicians, seven declined due to time constraints. Recruitment and concurrent data analysis continued until thematic saturation, resulting in a final sample of 12 physicians (10 female, 2 male). Initial thematic saturation was observed at the 9th interview. Applying a predefined stopping rule of three consecutive interviews confirming no new codes or conceptual insights (interviews 10, 11, and 12), data collection was formally concluded at a final sample size of 12 participants (*n* = 12). Consequently, the remaining three prospective participants were not interviewed. Participants represented an experienced cohort, with a median professional experience of 28.5 years (range: 7–35 years). They practiced in high-volume settings; the median number of average daily patient contacts per physician was 75.0 (IQR: 68.8–80.0), ranging from 60 to 100 patients per day (Table 1).

**Table 1:** Demographic and professional characteristics of participating family physicians (*N* = 12).

Characteristic	Value
Gender, *n* (%)	
Female	10 (83.3%)
Male	2 (16.7%)
Age (years)	
Median (IQR)	54.0 (37.8–56.3)
Range	35–58
Total Professional Experience (years)	
Median (IQR)	28.5 (11.5–32.3)
Range	7–35
Daily Patient Volume (Average patients/day)	
Median (IQR)	75.0 (68.8–80.0)
Range	60–100
Specialization, *n* (%)	
General Practitioner	9 (75.0%)
Family Medicine Specialist	3 (25.0%)

Thematic analysis of family physicians' transcripts revealed three overarching themes and subthemes mapped deductively to the seven domains of the TFA (Figure 1).

**Figure 1:**
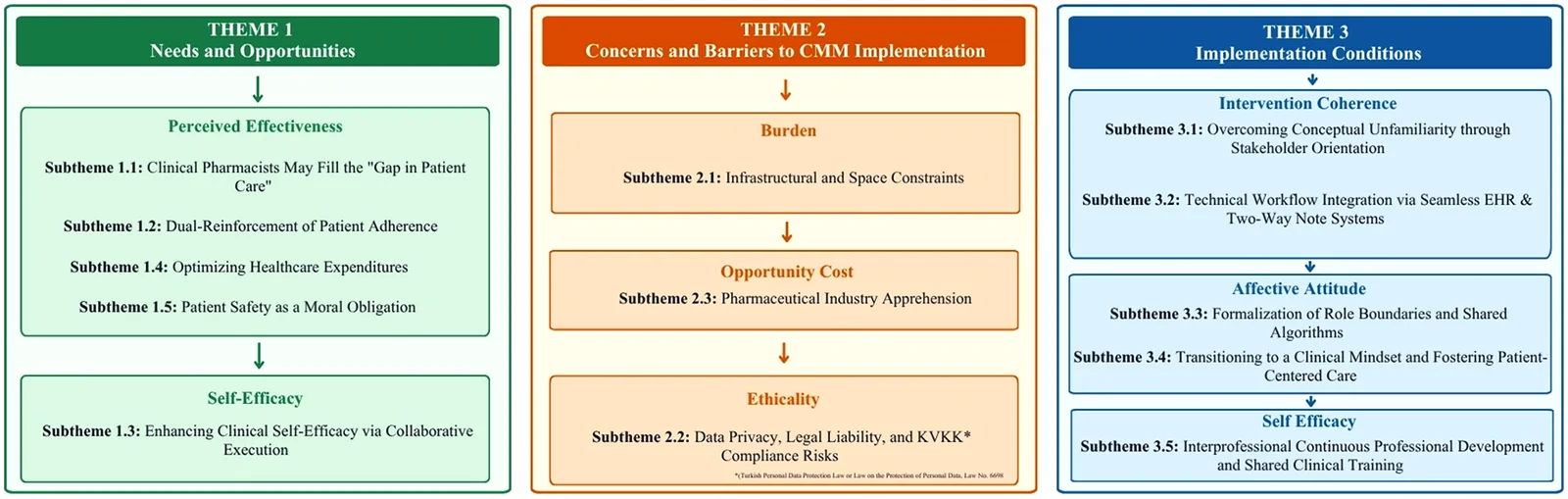
Summary of themes and subthemes mapped to the three overarching thematic pathways to their corresponding theoretical framework of acceptability (TFA) domains.

Theme 1 (Needs and Opportunities) captures clinical drivers, workload relief, and patient medication adherence benefits. Theme 2 (Concerns and Barriers to CMM Implementation) highlights macro-level structural, legal, and systemic obstacles that require external policy, capital, or governmental intervention. Theme 3 (Implementation Conditions) details actionable, local implementation enablers and operational conditions required to drive physician acceptance and collaborative success.

Overall, participants supported pharmacist-led CMM while outlining the systemic prerequisites and workflow adaptations necessary for successful deployment. A comprehensive matrix detailing the full coding tree—including all themes, TFA domains, specific subthemes, and extended participant quotes—is provided in Supplementary Material S3 (Comprehensive Qualitative Analysis Matrix TFA Framework).

### Theme 1. Needs and opportunities

This theme explains why family physicians believe a pharmacist-led medication review service is needed in primary care and the benefits they anticipate from collaborating with a pharmacist. It directly aligns with the TFA domains: Perceived Effectiveness (Subthemes 1.1, 1.2, 1.4, 1.5) and Self-Efficacy (Subtheme 1.3).

“*Clinical pharmacists may fill the gap in patient care” (Subtheme 1.1*): Family physicians highlighted a systemic gap in patient medication counseling, noting that extreme time constraints prevent them from conducting comprehensive reviews. They viewed the clinical pharmacist as a highly effective partner to fill this gap, estimating a substantial reduction in their overall clinical workload:

“First, the workload decreases, second. I can spend more time on more patients; I can be more productive. Informing the patient about adherence.. is also an extra time loss for us.. Pharmacists could fill that gap.” (P10, female, 50 yo, 25 years of experience).

*Dual-Reinforcement of Patient Medication Adherence (Subtheme 1.2):* Participants anticipated that clinical collaboration would establish a powerful reinforcement mechanism for patients. Hearing consistent medical and therapeutic advice from both a physician and a pharmacist was expected to significantly enhance patient compliance and trust:

“If the warning comes from two places at once—about vaccines, quitting smoking, exercising, or using medications properly—when the person receives reinforcement from two different sources, the effect increases.” (P7, male, 51 yo, 28 years of experience).

*Enhancing Clinical Self-Efficacy* via *Collaborative Execution (Subtheme 1.3):* Family physicians reported that extremely high daily patient volumes and the resulting cognitive fatigue directly impair their behavioral capacity to provide thorough, individualized medication management. Rather than viewing knowledge limits or fatigue as isolated emotional states, physicians linked them to structural barriers that compromise their clinical performance—deficits that collaborative Comprehensive Medication Management (CMM) is expected to ameliorate:

“Time, conditions, and workload don’t allow for many things. I can’t answer some questions, they ask about milligrams… when the patient sometimes asks if they should take this medication between this and that hour, I give round answers, and in that way, actually, I should be doing this or I should know this.. I’m talking about individualized treatment.” (P9, female, 38 yo, 12 years of experience).

By introducing a competent peer to address these temporal and cognitive deficits, physicians demonstrated strong behavioral readiness to operationalize joint clinical reviews, positioning the clinical pharmacist as an indispensable “second pair of eyes” to intercept polypharmacy risks:

“Unlike abroad, we don’t work with a very isolated 15 patients; the average patient is 70–80, so I can’t spend that much time in the polyclinic. Having this time that I can’t spend given by a different professional with similar competence would make my job much easier.” (P11, male, 35 yo, 12 years of experience).

“*As a second pair of eyes, I think a competent person sharing the clinic with us and being a partner in this treatment would increase quality and speed it up.”* (P6, female, 37 yo, 7 years of experience).

*Optimizing Healthcare Expenditures (Subtheme 1.4):* From a macro-level perspective, participants projected that CMM would reduce medication waste, eliminate redundant specialist visits for simple prescription renewals, and decrease long-term complication rates:

“In a way, it could be beneficial in reducing healthcare costs in the most positive sense… the patient’s complications will be fewer, healthcare expenditures will decrease, and the patient’s expected lifespan could increase.” (P11, male, 35 yo, 12 years of experience).

*Patient Safety as a Moral Obligation (Subtheme 1.5):* Physicians framed the integration of pharmacists as a moral imperative. Under the principle of *primum non nocere* (first, do no harm), protecting patients from adverse drug events superseded economic considerations:

“And more importantly, we would be applying the principle of do no harm. I mean, the economy is important, of course, but what is more important is do no harm.. our pharmacist friends.. make such a contribution to us, not only to the physician, but actually to the society as well.” (P1, female, 37 yo, 10 years of experience).

### Theme 2. Concerns and barriers to CMM implementation

Despite their overall enthusiasm, family physicians identified formidable structural, legal, and macro-level barriers that impede implementation within the existing healthcare system and necessitate coordinated policy or governmental intervention. This theme maps directly to the TFA domains of Burden (subthemes 2.1), Ethicality (subtheme 2.2), and Opportunity Cost (subtheme 2.3).

*Infrastructural and Space Constraints (Subtheme 2.1):* The most frequently reported barrier was a lack of physical infrastructure. Family physicians emphasized that FHCs are often located in converted apartments that cannot accommodate another desk or professional:

“Therefore, since sufficient physical area conditions are not provided.. I think the biggest difficulty is physical conditions, I mean, mechanically, there is no room for that pharmacist to stay there.” (P11, male, 35 yo, 12 years of experience).

*Data Privacy, Legal Liability, and KVKK Compliance Risks (Subtheme 2.2):* Strict legal concerns regarding medical data privacy under Türkiye's Personal Data Protection Law (KVKK) were raised. Physicians feared that non-professional auxiliary staff (such as pharmacy apprentices) might access confidential patient records if data flows are improperly secured outside clinical walls:

“Sharing the information.. constitutes a very big risk.. if the pharmacist shares this.. with non-pharmacist apprentices they employ, this constitutes a very big risk and danger..” (P12, female, 57 yo, 33 years of experience).

Some viewed this as an absolute legal and ethical red line:

“..sharing any note about the patient with someone else, even if it is another doctor friend, is not very appropriate either legally or ethically. .. I think it is not very ethical for the pharmacist to access the file between the patient and me, it’s not legal either..” (P11, male, 35 yo, 12 years of experience).

*Pharmaceutical Industry Apprehension (Subtheme 2.3):* An external, system-level economic barrier was also highlighted: the profit-driven motives of pharmaceutical manufacturers and depots. Because CMM champions rational drug use and curbs unnecessary polypharmacy, a participant expressed that corporate resistance may emerge against reduced drug sales volumes.

“I mean, can you imagine, you will work in the public sector, where will that Company A, Company B depot make money from? … For this system you mentioned to be implemented, it means you’re putting a stick in their wheel. … I mean, how can they make a crazy profit through you, through pharmacists, right now?” (P1, female, 37 yo, 10 years of experience)

### Theme 3. Implementation conditions

To facilitate CMM adoption and ensure sustainable collaboration, participants outlined actionable, local implementation conditions, workflow designs, and professional prerequisites. This theme maps directly to the TFA domains of Intervention Coherence (subthemes 3.1 and 3.2), Affective Attitude (subthemes 3.3 and 3.4), and Self-Efficacy (subtheme 3.5).

*Overcoming Conceptual Unfamiliarity through Stakeholder Orientation (Subtheme 3.1):* A foundational cognitive hurdle was initial unfamiliarity with CMM as an outpatient clinical service. Because formal clinical pharmacy is absent from Turkish FHCs, physicians initially perceived it as an abstract academic concept rather than an actionable workflow:

“No, I’ve never had any experience. So actually, we can say I’ve heard of it more as a book concept.” (P4, female, 54 yo, 32 years of experience)

*3.2):* Electronic Health Records (EHRs) are currently siloed. Physicians stressed that collaboration is contingent upon building integrated software infrastructure featuring a shared, bidirectional note-taking module to prevent administrative duplication:

“If that were the case, of course, there would be no time loss in collaboration.. and if there was something we missed with the notes the pharmacist made.. we would all see it from the same common system.” (P9, female, 38 yo, 12 years of experience).

*Formalization of Role Boundaries and Shared Algorithms (Subtheme 3.3):* Physicians insisted that clinical pharmacists must operate within clearly legislated boundaries that complement—rather than infringe upon—the physician's diagnostic and prescriptive authority:

“I would see it as a complementary collaboration, as long as the pharmacist does not go beyond the physician’s suggestions.” (P2, female, 56 yo, 31 years of experience).

Participants visualized interprofessional harmony through structured frameworks:

“Doctors would be nice with a system that feeds each other.. In summary, I mean that if everyone’s boundaries are clear, everyone can make mistakes, but we can correct each other’s mistakes with feedback.” (P7, male, 51 yo, 28 years of experience).

*Transitioning to a Clinical Mindset and Fostering Patient-Centered Care (Subtheme 3.4):* Family physicians expressed a desire for pharmacists to prioritize patient care, moving away from the “commercial” side of their trade and returning to a patient-centered healthcare role.

“I have never seen such an ethical understanding or a pro-patient attitude among pharmacist friends.. they generally act by thinking more from a commercial and financial perspective..” (P10, female, 50 yo, 25 years of experience).

*Interprofessional Continuous Professional Development and Shared Clinical Training (Subtheme 3.5):* Participants emphasized that successful CMM implementation requires structured continuing professional development (CPD) and joint training spanning *both* professions. While clinical pharmacists were noted to need enhanced training in patient communication and collaborative practice, physicians stressed that doctors and pharmacists must train together on shared clinical algorithms to bridge knowledge gaps and ensure they “speak the same language”:

“Both the doctor and the pharmacist must be trained in the same algorithm and speak the same language… We often don’t speak the same language, family physicians and pharmacists… we need to develop an algorithm so that we all speak the same language, approach it from the same scientific perspective.” (P8, female, 58 yo, 33 years of experience).

### Divergent case analysis: generational variations in acceptability

A comparison of transcripts within subgroups showed clear differences in perceived acceptability between early- to mid-career physicians and those later in their careers (see Table 2). While early- to mid-career physicians demonstrated higher prospective self-efficacy and intervention coherence, their late-career peers placed greater emphasis on immediate operational burdens and macro-level structural opportunity costs.

**Table 2:** Framework matrix of variations in prospective acceptability by career stage.

Identified theme	Corresponding TFA domains	Early-to-mid career physicians (7–15 years of experience, *n* = 4)	Late-career/established physicians (25+ years of experience, *n* = 8)
Theme 1: Needs & Opportunities	Perceived Effectiveness, Self−Efficacy, Opportunity Cost	High belief in clinical benefits; expressed strong self−efficacy regarding collaborative workflows; viewed coordination time as a manageable investment. *“No, if there is collaboration, nothing will happen. It will reduce the workload a lot.”* (P9, female, 38 yo, 12 years of experience)	High belief in clinical benefits; expressed cautious or conditionally dependent self−efficacy; viewed coordination time as a substantial opportunity cost. “*Explaining it one by one or even understanding that the patient understood it is a process for us..* *we can also leave those to you.” (*P10, female, 50 yo, 25 years of experience)
Theme 2: Concerns and Barriers	Burden, Ethicality	Anticipated minor−to−moderate workflow adjustments; emphasized digital integration. Ethically, expressed concerns regarding data privacy but generally viewed them as solvable through proper EHR integration. “*I think it is not very ethical for the pharmacist to access the file between me and the patient, it’s not legal either..”* (P11, male, 35 yo, 12 years of experience)	Anticipated significant operational and administrative burden; highlighted severe physical space constraints in clinics. Ethically, emphasized legal liability and data privacy vulnerabilities as severe operational barriers. “*If the pharmacist shares this shared information with the non−pharmacist apprentices they employ, this constitutes a very big risk and danger..”* (P12, female, 57 yo, 33 years of experience)
Theme 3: Implementation Conditions	Affective Attitude, Intervention Coherence	Expressed an optimistic and active outlook; demonstrated high conceptual familiarity and theoretical coherence regarding the clinical role of CMM. *“I would 100% support it… I wish it would happen.”* (P11, male, 35 yo, 12 years of experience)	Expressed a receptive but highly cautious outlook; demonstrated limited baseline familiarity with CMM, exhibiting conceptual gaps in practical execution. “*I understand, so I support it 60%, I have reservations for 40% due to this personal disagreement problem.”* (P12, female, 57 yo, 33 years of experience)

## Discussion

This study provides a theory-driven evaluation of the prospective acceptability of pharmacist-led CMM within Turkish primary care. The findings reveal a distinct implementation paradox: while family physicians strongly endorse CMM for its clinical effectiveness, workload relief, and collaborative self-efficacy (Theme 1), prospective uptake is contingent upon navigating severe macro-level structural and systemic barriers requiring external reform (Theme 2), alongside the establishment of actionable implementation conditions, digital integration, and formal role boundaries (Theme 3). Evaluated through the TFA, this specific configuration indicates that while CMM is conceptually valued and morally endorsed, it remains operationally precarious in existing primary care settings (29).

Our results suggest that Turkish family physicians view clinical pharmacists not as professional competitors but as a vital clinical safety net that can fill an acute patient-care vacuum (Subtheme 1.1). A participant projected that an integrated pharmacist could handle 30% to 40% of routine therapeutic workload. This workflow relief directly addresses the “triple burden” of primary care—cognitive load, time pressure, and administrative strain—well documented in the global literature (30). While general CMM models consistently report qualitative reductions in physician burnout (31), targeted studies demonstrate that integrated pharmacists can effectively manage a high volume of routine therapeutic tasks, including refill processing and safety monitoring (32).

Crucially, our findings extend the operationalization of Self-Efficacy (Subtheme 1.3: Enhancing Clinical Self-Efficacy via Collaborative Execution) within interprofessional care models (24). In Sekhon et al.'s foundational framework, self-efficacy is an individual's confidence in performing required intervention behaviors (22). In our study, context-driven adaptation shows that cognitive fatigue, time constraints, and extreme daily patient volumes directly undermine family physicians’ confidence in independently conducting detailed, individualized pharmacotherapy reviews. Integrating clinical pharmacists serves as an external cognitive enabler, restoring and sustaining physicians' self-efficacy in delivering high-quality care. At the same time, physicians expected broader benefits from optimizing healthcare expenditures (Subtheme 1.4) and firmly regarded patient safety as a moral obligation (Subtheme 1.5).

Instead of showcasing professional protectionism or territorial identity (33), family physicians embraced a “complementary collaboration” approach, expecting a synergistic dual-reinforcement mechanism (Subtheme 1.2). Using metaphors like the “four legs of a table” or a “second pair of eyes,” clinicians noted that patients receiving synchronized guidance from both doctors and pharmacists were more likely to adhere to regimens and trust recommendations (9).

However, prospective acceptability was substantially tempered by external, legal, and structural barriers (Theme 2: Concerns and Barriers to CMM Implementation), which require substantial governmental support, capital investment, and legislative reform. Providers highlighted severe infrastructure and physical space constraints in converted clinic buildings (Subtheme 2.1), stringent data privacy, legal liability, and KVKK compliance risks related to auxiliary non-professional staff (Subtheme 2.2), and external commercial apprehension from the pharmaceutical industry (Subtheme 2.3).

Addressing initial conceptual ambiguity and unfamiliarity with CMM as an abstract academic concept—which constitutes an initial cognitive hurdle (Subtheme 3.1)—represents the first foundational prerequisite for Theme 3 (Implementation Conditions). Achieving full operational coherence requires resolving fragmented EHR and software architectures through seamless, bidirectional note-taking integration (Subtheme 3.2). Consequently, physicians' positive Affective Attitude toward prospective adoption remains conditional on establishing clear operational prerequisites: formalizing role boundaries and shared clinical algorithms (Subtheme 3.3), transitioning from a traditional “commercial” mindset (34) to a strictly patient-centered ethical framework (Subtheme 3.4), and establishing joint interprofessional continuous professional development (Subtheme 3.5).

Achieving this long-term paradigm shift inherently requires foundational reforms to undergraduate pharmacy curricula. By pivoting away from product-dispensing models and embedding interprofessional education (IPE), clinical rotations, and direct patient-care competencies early in the academic pathway, pharmacy schools can produce a workforce naturally equipped for CMM (35). Recent quantitative evidence from China strongly supports this local demand for structured educational integration, showing that participating in joint continuing education and establishing interprofessional communication during residency training significantly increase the frequency and positivity of physician-pharmacist collaboration (36).

Importantly, our findings highlighted serious legal and ethical concerns about access to patient data and confidentiality under Türkiye's Personal Data Protection Law (KVKK compliance) (Subtheme 2.2), particularly the risk of unauthorized exposure by non-professional staff (such as pharmacy apprentices). These provider concerns reflect an accurate understanding of the current legal framework: national health records (*e-Nabız*) strictly limit access to clinical data to treating physicians, while community pharmacists interact solely through the billing-focused *Medula* platform. Therefore, establishing CMM requires health authorities to implement formal data governance protocols and tiered, audited EHR access profiles.

Generational dynamics further shaped prospective acceptability. Younger practitioners (mean age ∼38) exhibited an “innovation-friendly” profile, with high baseline readiness for collaborative workflows. Conversely, senior clinicians (mean age ∼55) prioritized operational burden and data privacy. Although international studies from Kuwait, England, and Ireland reflect these patterns—suggesting that younger clinicians are more receptive to integration and older practitioners are more cautious—well-organized evidence definitively supporting these age-related differences remains insufficient (37–39). In fact, large-scale evidence from China shows that older family physicians with more years of clinical practice are significantly more likely to share treatment goals, express willingness to collaborate, and adjust patients' medications based on pharmacists' advice (36). Because these inconsistent international findings often rely on qualitative designs with small sample sizes, they likely reflect specific cross-cultural professional dynamics rather than universal principles.

To bridge this generational divide and facilitate the transition from conceptual agreement to tangible behavioral change, we propose four actionable implementation pillars with pragmatic recommendations for policymakers, healthcare administrators, and professional bodies (40–42):

1. Legislative and Policy Reform (*For Policymakers & Ministry of Health*): Enact formal Collaborative Practice Agreements (CPAs) within Turkish health legislation to legally authorize clinical pharmacists to provide CMM services, order routine monitoring tests, and adjust therapy for defined chronic conditions. In parallel, establish sustainable reimbursement and public contracting mechanisms for clinical pharmacy services under SGK.
2. Infrastructural and Digital Integration (*For Healthcare Administrators & IT Regulators*): Standardize Family Health Center infrastructure guidelines to require designated private consultation areas for clinical pharmacists. Simultaneously, build a dedicated, role-based primary care CMM module within *e-Nabız* that enables bidirectional clinical note sharing between physicians and pharmacists while maintaining strict KVKK compliance and audit trails.
3. Educational Curriculum Alignment (*For Pharmacy/Medical Faculties & Professional Chambers*): Restructure undergraduate pharmacy curricula to embed mandatory clinical clerkships, therapeutic communication, and joint Interprofessional Education (IPE) with medical students (35, 36). The Turkish Pharmacists’ Association (TEB) and the Turkish Medical Association (TTB) should co-develop accredited Continuous Professional Development (CPD) modules to establish shared clinical algorithms and clarify roles.
4. Strategic Champion-Led Pilots (*For District Health Directorates & Clinic Leads*): Roll out iterative, localized pilot CMM programs, led by younger “clinical champions,” at selected Family Health Centers. These pilots should focus on high-risk polypharmacy and multimorbid older adult cohorts to generate empirical safety and workflow data, resolve interprofessional friction, and demonstrate operational feasibility to senior practitioners before nationwide scaling.

### Limitations of the study

While this study establishes a rigorous baseline by being the first to apply the TFA to primary care pharmacy integration in Türkiye, several limitations remain.

First, because this qualitative study uses a purposive sample of 12 family physicians, the findings offer in-depth thematic insights but are not statistically generalizable to the entire primary care physician population in Türkiye. Additionally, our sample has a demographic gap in career stage: we did not recruit any physicians with 16 to 24 years of experience, leaving this mid-career transition cohort underrepresented.

Second, because it is prospective, this study captures anticipated rather than experienced acceptability of a hypothetical service model. This creates an inherent “intention-behavior gap,” in which high verbal endorsement may falter when confronted with real-world clinical workloads and day-to-day coordination challenges.

Third, because CMM is not yet integrated into the Turkish healthcare system, most participants lacked baseline familiarity with the concept before the interview. Because their understanding relied on explanatory definitions and hypothetical scenarios introduced during data collection, framing effects and social desirability bias are risks; participants may have expressed support to align with perceived academic or professional ideals rather than with operational realities.

Fourth, this study captured only primary care physicians' perspectives. The viewpoints of other essential stakeholders—namely community and clinical pharmacists, patients, healthcare administrators, and policymakers—were not evaluated, which is critical to a complete assessment of multi-level implementation feasibility.

Finally, our sample was predominantly female (83.3%) and exclusively urban (conducted in Istanbul), which may obscure distinct resource constraints, infrastructural variations, or differing interprofessional dynamics present in rural or underserved Turkish primary care settings.

## Conclusion and policy implications

This qualitative study shows that pharmacist-led CMM has strong prospective acceptability among urban family physicians in Türkiye. Moving away from traditional professional protectionism or territorial boundary guarding, physicians view the clinical pharmacist not as a threat to medical authority but as an indispensable clinical safety net capable of filling an acute care vacuum. Amid the crushing operational pressures of hyper-accelerated polyclinic volumes, integrating a dedicated pharmacotherapy expert is strongly endorsed to mitigate physician cognitive fatigue, strengthen patient medication adherence through dual reinforcement loops, and restore team-based collaborative self-efficacy. Ultimately, while CMM is highly desired and conceptually well developed, its real-world implementation remains operationally precarious until health authorities actively bridge the divide between systemic willingness and infrastructural reality.

### Practice and policy implications

– *Legislative and Financial Formalization:* Health authorities must incorporate formal Collaborative Practice Agreements (CPAs) into Turkish health legislation to legally authorize clinical pharmacists to conduct medication reviews and adjust therapy within defined chronic parameters. Sustainable public contracting and reimbursement mechanisms managed by the Social Security Institution (SGK) must support this.
– *Digital Governance and Infrastructure Tiering:* IT regulators must build a dedicated, role-based primary care CMM module for *e-Nabız*. This module must enable secure, bidirectional note-sharing between physicians and pharmacists while enforcing strict, automated data-masking protocols to prevent unauthorized exposure of data to non-professional pharmacy apprentices. At the same time, physical clinic building guidelines must be standardized to mandate designated private clinical consultation spaces.
– *Curricular Alignment and Cultural Shift:* The Higher Education Council should mandate structural reforms to undergraduate pharmacy curricula, including mandatory clinical clerkships and joint Interprofessional Education (IPE) modules with medical students to establish mutual role clarity early. In parallel, professional chambers (TEB and TTB) must co-develop accredited continuing education frameworks to shift the neighborhood pharmacy’s identity from a commercial storefront to a trusted medical institution.

## Data Availability

The datasets generated and analyzed during this study, including the thematic framework, coding scheme and representative verbatim quotations, are included in the article and its Supplementary Material. Raw interview recordings and unedited transcripts cannot be shared because of participant confidentiality and institutional ethics restrictions. Further inquiries can be directed to the corresponding author.
