# Supplementary material for: Family physicians’ acceptability, perceived value, and barriers of pharmacist-led medication management in primary care—a theory-based qualitative study
Source: Front Med (Lausanne). 2026 Oct 28;13:1949962. doi: 10.3389/fmed.2026.1949962 (PMC13752058; doi:10.3389/fmed.2026.1949962)
Supplement: Supplementary file 1 [file Table1.docx]

**Supplemental Material:**

***Supplement 01.*** *Questionnaire with TFA Domain Alignment*

| **No** | **Interview Question** | **TFA Domain(s)** |
| --- | --- | --- |
| 1 | What role do pharmacists play in the healthcare system beyond just preparing prescriptions? | Intervention Coherence |
| 2 | Have you ever heard of concepts like “comprehensive medication management” or “medication review”? | Intervention Coherence |
| 3 | Within the scope of comprehensive medication management, services that clinical pharmacists can offer in collaboration with physicians, with priority given to multimorbid and/or patients who use a lot of medication, include; disease management, patient education and counseling, planning and management of drug therapy, regular review of drug therapy, prevention and resolution of drug-related problems, supporting patient adherence to treatment, and evaluation of treatment outcomes. Do you have knowledge about these service headings? If yes, which ones do you know or have you experienced? If no, in your opinion, why might there not be enough awareness about these services? | Intervention Coherence, Affective Attitude |
| 4 | Are pharmacists qualified to provide these services I listed above? | Affective Attitude |
| 5 | Do you think the services I mentioned above can be implemented in family health centers? | Perceived Effectiveness, Burden |
| 6 | As a healthcare professional working in primary healthcare services, would you support pharmacists providing pharmaceutical care services such as comprehensive medication management within a framework of collaboration with doctors? If you support it, why? If you don't support it, what would your reservations be? | Self-efficacy Affective Attitude, Opportunity Costs |
| 7 | What are the potential benefits and possible risks of these services offered by pharmacists? | Perceived Effectiveness, Burden |
| 8 | If pharmacists provide comprehensive medication management services in collaboration with doctors, would you want to communicate with pharmacists through electronic patient files? | Burden, Intervention Coherence, Self-efficacy |
| 9 | Would you find it appropriate for the pharmacist to leave notes in these files or to see existing notes? | Ethicality, Burden |
| 10 | What are the biggest obstacle(s) standing in the way of these services? | Burden, Opportunity Costs |
| 11 | What are the existing fundamental opportunities for the implementation of pharmaceutical care services in family health centers in Türkiye? | Intervention Coherence |
| 12 | Do you see the pharmacist’s contribution to the treatment process and their responsibilities within the scope of comprehensive medication management as an interference with your professional field or as a complementary collaboration? | Ethicality, Affective Attitude |
| 13 | Do these contributions of the pharmacist reduce the workload of physicians, bring an extra coordination burden, or have no effect? | Burden, Perceived Effectiveness |
| 14 | Would a pharmacist’s direct seeing of patients in a family health center affect the patient-physician relationship positively or negatively in terms of the patient’s trust? Could it create information confusion or an authority conflict for the patient? | Ethicality, Affective Attitude |
| 15 | Do you have any additional thoughts, suggestions, or comments you would like to add regarding comprehensive medication management, pharmaceutical care, or pharmacist-physician collaboration that were not addressed in this interview? | Open to all domains |
