# Supplementary material for: Family physicians’ acceptability, perceived value, and barriers of pharmacist-led medication management in primary care—a theory-based qualitative study
Source: Front Med (Lausanne). 2026 Oct 28;13:1949962. doi: 10.3389/fmed.2026.1949962 (PMC13752058; doi:10.3389/fmed.2026.1949962)
Supplement: Supplementary file 2 [file Table2.docx]

**Supplemental Material:**

***Supplement 02.***  *COREQ (Consolidated Criteria for Reporting Qualitative Research) Checklist Compliance Report*

Study Title: Exploring the Acceptability of Clinical Pharmacist-Led Comprehensive Medication Management in Turkish Primary Healthcare Centers: A Qualitative Study with Family Physicians

This table presents a detailed evaluation of the COREQ checklist across 32 items, grouped into three domains. Each item is assessed for its presence and clarity in the manuscript, with corresponding comments.

| ***Item*** | ***COREQ Question*** | ***Response*** | ***Compliance*** |
| --- | --- | --- | --- |
| **Domain 1: Research team and reflexivity** | | | |
| 1 | Interviewer/facilitator | Lead researcher conducted all interviews via Zoom. | ✅ |
| 2 | Credentials | Researcher held a PhD in Clinical Pharmacy. | ✅ |
| 3 | Occupation | Researcher was a university faculty member at the time of study. | ✅ |
| 4 | Gender | Interviewer identified as female. | ✅ |
| 5 | Experience and training | Experienced in qualitative research and pharmacy practice. | ✅ |
| 6 | Relationship established | Contact established prior to interviews via phone; rapport built before data collection. | ✅ |
| 7 | Participant knowledge of interviewer | Participants were informed of the study purpose and interviewer’s role. | ✅ |
| 8 | Interviewer characteristics | Researcher’s pharmacy background noted; reflexivity discussed. | ✅ |
| **Domain 2: Study Design** | | | |
| 9 | Methodological orientation | Thematic analysis guided by Theoretical Framework of Acceptability (TFA). | ✅ |
| 10 | Sampling | Purposive sampling with maximum variation (age, experience, region). | ✅ |
| 11 | Method of approach | Telephone invitations followed by Zoom interviews. | ✅ |
| 12 | Sample size | 12 participants until data saturation achieved. | ✅ |
| 13 | Non-participation | 7 out of 22 invited physicians declined due to time constraints (stated in Methods and Results). | ✅ |
| 14 | Setting of data collection | Zoom interviews conducted in private professional settings. | ✅ |
| 15 | Presence of non-participants | Only interviewer and participant were present. | ✅ |
| 16 | Description of sample | Demographic table provided (sex, age, experience, specialty). | ✅ |
| 17 | Interview guide | Semi-structured guide aligned with TFA domains included. | ✅ |
| 18 | Repeat interviews | No repeat interviews conducted. | ✅ |
| 19 | Audio/visual recording | Audio recordings obtained with consent. | ✅ |
| 20 | Field notes | Field notes not taken after interviews to capture context. | ⚠️ |
| 21 | Duration | Interviews lasted for an average of 23 minutes (range: 13–37 minutes). | ✅ |
| 22 | Data saturation | Data collection ceased after saturation was achieved. | ✅ |
| 23 | Transcripts returned | Transcripts not returned to participants for feedback. | ⚠️ |

| **Domain 3: Analysis and Findings** | | | |
| --- | --- | --- | --- |
| 24 | Number of data coders | Two researchers (ZIS and AA) coded data independently using a bilingual approach (Turkish and English transcripts); discrepancies were resolved by discussion | ✅ |
| 25 | Description of coding tree | Coding structure followed TFA domains; summarized descriptively. | ✅ |
| 26 | Derivation of themes | Themes derived deductively from TFA and inductively from data. | ✅ |
| 27 | Software | NVivo used for data management and coding. | ✅ |
| 28 | Participant checking | Members verified the accuracy of the transcripts and endorsed the final themes | ✅ |
| 29 | Quotations presented | Direct quotations from participants included with ID codes. | ✅ |
| 30 | Data and findings consistent | Findings clearly supported by data excerpts. | ✅ |
| 31 | Clarity of major themes | Major themes clearly identified per TFA domain. | ✅ |
| 32 | Clarity of minor themes | Minor variations discussed within main themes. | ✅ |

**Summary of COREQ Compliance**

| Total Items | 32 |
| --- | --- |
| Fully Met (✅) | 30 |
| Partially Met (⚠️) | 2 |
| Not Met (❌) | 0 |

**Overall COREQ compliance score: 25/32 = ~ 94%**

*Interpretation:* High reporting quality. The study meets most COREQ criteria, particularly in methodology transparency, reflexivity, and thematic rigor, with minor limitations in participant validation of their individual transcripts.
