# Supplementary material for: Family physicians’ acceptability, perceived value, and barriers of pharmacist-led medication management in primary care—a theory-based qualitative study
Source: Front Med (Lausanne). 2026 Oct 28;13:1949962. doi: 10.3389/fmed.2026.1949962 (PMC13752058; doi:10.3389/fmed.2026.1949962)
Supplement: Supplementary file 3 [file Table3.docx]

**Supplemental Material:**

**Supplement 03.** Qualitative interview themes, subthemes, codes and example quote from healthcare providers' interviews

| **Theme** | **TFA Domain** | **Subtheme** | **Quotes** | **Codes** |
| --- | --- | --- | --- | --- |
| Needs and Opportunities | *Perceived Effectiveness* | Clinical Pharmacists May Fill the "Gap in Patient Care"  (Subtheme 1.1) | *“It would reduce our workload... by 30-40 percent.”* (P9, female, 38 yo, 12 years of experience)  *“First, the workload decreases, second... I can spend more time on more patients; I can be more productive. Informing the patient about adherence... is also an extra time loss for us... Pharmacists could fill that gap.”* (P10, female, 50 yo, 25 years of experience). | Workload reduction,  Filling care gap,  Timesaving,  Increased productivity |
|  |  | Dual-Reinforcement of Patient Adherence (Subtheme 1.2) | *“If the warning comes from two places at once—about vaccines, quitting smoking, exercising, or using medications properly—when the person receives reinforcement from two different sources, the effect increases.”* (P7, male, 51 yo, 28 years of experience).  *“If information such as the effectiveness, complications, and incompatibilities of medications is detailed by a pharmacist, I think the trust in the physician, the trust in the medication, and the trust in science will increase even more.”* (P6, female, 37 yo, 7 years of experience). |  |
|  |  | Optimizing Healthcare Expenditures  (Subtheme 1.4) | *“Well, so as far as I understand, significant savings can be made through what we call healthcare economics this way as well.*” (P1, female, 37 yo, 10 years of experience).  *“In a way, it could be beneficial in reducing healthcare costs in the most positive sense… the patient’s complications will be fewer, healthcare expenditures will decrease, and the patient’s expected lifespan could increase.”* (P11, male, 35 yo, 12 years of experience). | Healthcare cost reduction,  Waste reduction,  Decreased long-term complications |
|  |  | Patient Safety as a Moral Obligation (Subtheme 1.5) | *“And more importantly, we would be applying the principle of do no harm. I mean, the economy is important, of course, but what is more important is do no harm... our pharmacist friends... make such a contribution to us, not only to the physician, but actually to the society as well.”* (P1, female, 37 yo, 10 years of experience). | Primum non noncore,  Moral imperative,  Patient safety superseding economic costs |
|  | *Self-Efficacy* | Enhancing Clinical Self-Efficacy via Collaborative Execution (Subtheme 1.3) | *“I do a great job in the morning, I'm active in the morning, I can make good adjustments, but after that my battery runs out, I can't do things anymore, I don't have time and energy left... I would like to do these things with someone who is conscious.”* (P5, female, 58 yo, 35 years of experience).  *“Time, conditions, and workload don't allow for many things. I can't answer some questions, they ask about milligrams… when the patient sometimes asks if they should take this medication between this and that hour, I give round answers, and in that way, actually, I should be doing this or I should know this... I'm talking about individualized treatment.”* (P9, female, 38 yo, 12 years of experience).  *“Unlike abroad, we don't work with a very isolated 15 patients; the average patient is 70-80, so I can't spend that much time in the polyclinic... Having this time that I can't spend given by a different professional with similar competence would make my job much easier.”* (P11, male, 35 yo, 12 years of experience).  *“As a second pair of eyes, I think a competent person sharing the clinic with us and being a partner in this treatment would increase quality and speed it up.” (P6, female, 37 yo, 7 years of experience).*  *“Pharmacists are trained in pharmacology and drug interactions; they are qualified to manage these aspects better than physicians.”* (P1, female, 37 yo, 10 years of experience). | Fatigue/burnout relief,  Coping with high patient volume (70–80/day),  Managing drug interactions,  "second pair of eyes" |
| Concerns and Barriers to CMM Implementation | *Ethicality* | Infrastructural and Space Constraints (Subtheme 2.1) | *“We provide primary care services in very bad buildings... even our nurses have a space problem. We can only give them one room... I think there may be a space problem.”* (P5, female, 58 yo, 35 years of experience)  *“Therefore, since sufficient physical area conditions are not provided... I think the biggest difficulty is physical conditions, I mean, mechanically, there is no room for that pharmacist to stay there.”* (P11, male, 35 yo, 12 years of experience).  *“Our biggest problem as family physicians is a space problem, an area problem… I think the first and biggest problem is the area problem; an investment needs to be made...”* (P6, female, 37 yo, 7 years of experience). | Physical space shortage,  Architectural inadequacy,  Lack of consultation rooms |
|  | *Burden* | Data Privacy, Legal Liability, and KVKK (GDPR) Compliance Risks (Subtheme 2.2) | *“Sharing the information... constitutes a very big risk... if the pharmacist shares this... with non-pharmacist apprentices they employ, this constitutes a very big risk and danger...”* (P12, female, 57 yo, 33 years of experience).  *“...sharing any note about the patient with someone else, even if it is another doctor friend, is not very appropriate either legally or ethically. ... I think it is not very ethical for the pharmacist to access the file between the patient and me, it's not legal either...”* (P11, male, 35 yo, 12 years of experience). | KVKK/GDPR compliance risks,  Apprentice data breach,  Confidentiality concerns |
|  | *Opportunity Cost* | Pharmaceutical Industry Apprehension (Subtheme 2.3) | *“I mean, can you imagine, you will work in the public sector, where will that Company A, Company B depot make money from? … For this system you mentioned to be implemented, it means you're putting a stick in their wheel. … I mean, how can they make a crazy profit through you, through pharmacists, right now?”* (P1, female, 37 yo, 10 years of experience) | Pharmaceutical industry resistance,  Commercial profit clash,  Rational use counter-incentives |
| Implementation Conditions | *Intervention Coherence* | Overcoming Conceptual Unfamiliarity through Stakeholder Orientation (Subtheme 3.1) | *"Comprehensive medication management, no... I haven't heard of it before"* (P9, female, 38 yo, 12 years of experience)  *"No, I've never had any experience. So actually, we can say I've heard of it more as a book concept."* (P4, female, 54 yo, 32 years of experience)  *"It seems like it can be inferred from the name, but it's something I don't understand, something that needs to be understood more"* (P6, female, 37 yo, 7 years of experience) | Conceptual unfamiliarity,  Abstract academic ideal,  Lack of mental models |
|  |  | Technical Workflow Integration via Seamless EHR & Two-Way Note Systems  (Subtheme 3.2) | *“But I think that depends a little on the skills of the software developers… if there is a place where we can also see the pharmacist's note in the form of small notes, this would not add a very, very big responsibility and burden to us…”* (P6, female, 37 yo, 7 years of experience).  *"If that were the case, of course, there would be no time loss in collaboration... and if there was something we missed with the notes the pharmacist made... we would all see it from the same common system.”* (P9, female, 38 yo, 12 years of experience). | EHR software integration,  Bidirectional note-taking module,  Digital workflow continuity |
|  | *Affective Attitude* | Formalization of Role Boundaries and Shared Algorithms (Subtheme 3.3) | *“Pharmacists can do this, but the system must recognize their authority formally.”* (P11, male, 35 yo, 12 years of experience).  *"I would see it as a complementary collaboration, as long as the pharmacist does not go beyond the physician's suggestions.”* (P2, female, 56 yo, 31 years of experience).  *“I always see the patient, pharmacist, doctor, and patient's relative as the four legs of a table, meaning there must be a meaningful, effective, and efficient collaboration between them. If there is a problem with one of them, a problem will arise somewhere... I see it as a complementary collaboration.”* (P2, female, 56 yo, 31 years of experience).  *“We need to develop an algorithm so that we all speak the same language.”* (P8, female, 58 yo, 33 years of experience).  *“Doctors would be nice with a system that feeds each other... In summary, I mean that if everyone's boundaries are clear, everyone can make mistakes, but we can correct each other's mistakes with feedback.”* (P7, male, 51 yo, 28 years of experience). | Defined professional boundaries,  Preserved prescribing authority,  Shared clinical algorithms |
|  | *Affective Attitude* | Transitioning to a Clinical Mindset and Fostering Patient-Centered Care  (Subtheme 3.4) | *“I have never seen such an ethical understanding or a pro-patient attitude among pharmacist friends... they generally act by thinking more from a commercial and financial perspective...”* (P10, female, 50 yo, 25 years of experience). | Commercial vs. clinical mindset, patient-centered shift, Ethical alignment |
|  | *Self-Efficacy* | Interprofessional Continuous Professional Development and Shared Clinical Training (Subtheme 3.5) | *“Both the doctor and the pharmacist must be trained in the same algorithm and speak the same language… We often don't speak the same language, family physicians and pharmacists… we need to develop an algorithm so that we all speak the same language, approach it from the same scientific perspective.”* (P8, female, 58 yo, 33 years of experience).  *“I believe pharmacists have the competence to provide medication reviews, but they may need additional training in patient communication.”* (P8, female, 58 yo, 33 years of experience)  *“I think they can provide it, of course, in line with their experience... by working jointly with the clinic, they can increase their experience...”* (P4, female, 54 yo, 32 years of experience)  *“By enriching the training, a little more maybe, the pharmacy education a little more. Continuous professional development is also done, I mean, I think it should be done, and there is a need for it. Some of them also work in pharmacies.”* (P7, male, 51 yo, 28 years of experience). | Joint CPD,  Interprofessional training,  Communication skill building,  Shared scientific perspective |
